# Immunoglobulin A-Dominant Post-Infectious Glomerulonephritis Mimicking Henoch-Schönlein Purpura Nephritis in a Patient With Methicillin-Sensitive Staphylococcus aureus Bacteremia

**DOI:** 10.7759/cureus.86409

**Published:** 2025-06-20

**Authors:** Victoria D Delk, Vishwajeeth Pasham, Kathleen McKinnon, Pranav Shah

**Affiliations:** 1 Internal Medicine, Medical University of South Carolina, Charleston, USA; 2 Pathology and Laboratory Medicine, Medical University of South Carolina, Charleston, USA; 3 Rheumatology, Medical University of South Carolina, Charleston, USA

**Keywords:** acute kidney injury, henoch schönlein purpura, methicillin-sensitive staphylococcus aureus, palpable purpura, post-infectious glomerulonephritis

## Abstract

Staphylococcal infections remain a prominent cause of hospital- and community-acquired infections in the United States. Glomerulonephritis with predominant Immunoglobulin A (IgA) deposition following staphylococcal infection has been described as IgA-dominant postinfectious glomerulonephritis (IgA-PIGN). This clinical entity can mimic Henoch-Schönlein purpura nephritis (HSPN) given that it may also be preceded by staphylococcal infection, have similar kidney biopsy findings, and present with similar signs and symptoms.

Our patient presented with a palpable purpuric rash and acute kidney injury following methicillin-sensitive *Staphylococcus aureus* (MSSA) bacteremia treated with intravenous cefazolin. A skin biopsy revealed leukocytoclastic vasculitis and vascular IgA deposition. Urinalysis showed active urinary sediment with hematuria and proteinuria. These findings were concerning for possible HSPN that was triggered by either staphylococcal infection or antibiotic exposure. Given worsening renal injury, the patient was started on empiric methylprednisone while awaiting kidney biopsy. When performed, the findings of diffuse proliferative glomerulonephritis with exudative features, subepithelial and mesangial deposits with strong C3 and moderate IgA staining were more consistent with IgA-PIGN over HSPN. Since IgA-PIGN is not responsive to steroids and may in fact be worsened by immunosuppression, methylprednisone was discontinued. The patient’s renal function improved; however, he developed multi-organism bacteremia and duodenal perforation, ultimately leading to death.

This case demonstrates the importance of distinguishing IgA-PIGN from HSPN in a patient with Staphylococcal infection presenting with renal injury and purpuric rash. Characteristic clinical signs and renal biopsy findings may aid in differentiating the two clinical entities so that the appropriate treatment can be initiated.

## Introduction

Staphylococcal infections account for significant morbidity and mortality in the United States. Although hospital- and community-acquired methicillin-resistant *Staphylococcus aureus* (MRSA) infections have decreased, methicillin-sensitive *S. aureus* (MSSA) infections remain a significant burden both in community and inpatient settings [1]. Glomerulonephritis associated with staphylococcal infections was initially described in 1980 by Spector et al., in which renal biopsies showed dominant and codominant Immunoglobulin A (IgA) deposits [2]. This was described in more detail in 1995 by Koyama et al., who reported 10 cases of glomerulonephritis following MRSA infection, with renal biopsies showing glomerular deposition of IgA, IgG, and C3 [3]. This clinical entity of acute kidney injury with biopsy showing proliferative glomerulonephritis and glomerular IgA and C3 deposition, often with mesangial or subepithelial humps seen on EM, has been named IgA-dominant postinfectious glomerulonephritis (IgA-PIGN) [4]. Subsequent studies have reported additional cases of IgA-PIGN associated with Staphylococcal infection, most commonly following skin, deep-seated abscesses, surgical wounds, pneumonia, and joint infection [4-14].

This IgA predominance is in contrast to the classic poststreptococcal PIGN, in which renal biopsies typically show mesangial or peripheral capillary wall deposition of IgG and C3 or C3 alone; IgA is typically absent [5]. It also poses a diagnostic challenge as the renal biopsy findings are similar to those seen in IgA nephropathy (IgAN) or Henoch-Schönlein purpura nephritis (HSPN), particularly since Staphylococcal infection has been described to precede Henoch-Schönlein purpura (HSP) with and without nephritis [4,5,10-19]. This is especially difficult when the patient has preexisting IgAN or presents with purpuric rash mimicking HSP, which may happen in 15-40% of patients with IgA-PIGN [6,11,14,20]. Distinguishing HSPN/IgAN and IgA-PIGN is essential because the treatment implications are different: While the former can be treated with immunosuppressive therapy, the latter may be exacerbated by immunosuppression even with co-treatment with antibiotics [12].

## Case presentation

Our patient was a 64-year-old-male with a past medical history of atrial fibrillation, hyperlipidemia, hypertension, type 2 diabetes, obstructive sleep apnea, and chronic venous insufficiency. During a previous hospitalization, the patient was found to have MSSA bacteremia complicated by left knee septic arthritis. He underwent a left knee washout and had an echocardiogram that showed no evidence of vegetations. Ultimately, no definitive primary source was determined, and he was discharged with a plan to continue IV cefazolin for four weeks of antibiotic therapy. The patient also remained on warfarin for anticoagulation in the setting of atrial fibrillation.

He presented again six days later complaining of a rash that began four days after discharge and 11 days after his initial positive blood cultures. He first noticed an area of erythema on his right arm near his central line insertion site, which then spread to his left arm and hands. He was found to have an international normalized ratio (INR) 9.5 and serum creatinine was elevated to 1.93 mg/dL from 0.97 mg/dL at recent discharge. Urinalysis was notable for new hematuria, pyuria, and proteinuria (urine protein/creatinine ratio 1.7 g/g). The patient's laboratory values are noted in Table 1.

**Table 1:** Laboratory values *Per high-power field BUN: Blood urea nitrogen; INR: International normalized ratio; ANA: Anti-nuclear antibody; c-ANCA: Cytoplasmic antineutrophil cytoplasmic antibody; p-ANCA: Perinuclear anti-neutrophil cytoplasmic antibody; MPO: Myeloperoxidase; Ab: Antibody; PR3: Proteinase-3; RF: Rheumatoid factor; dsDNA: double-stranded DNA; GBM: Glomerular basement membrane; IgG: Immunoglobulin G; HBsAg: Surface antigen; HBsAb: Surface antibody; HBcAb: Core antibody; HIV: Human immunodeficiency virus

Laboratory finding	Result	Reference
Creatinine		
Baseline	0.7-0.9 mg/dL	0.60-1.40 mg/dL
Day 1 (admission)	1.93 mg/dL	0.60-1.40 mg/dL
Day 11 (peak creatinine)	3.66 mg/dL	0.60-1.40 mg/dL
Day 36 (discharge)	1.14 mg/dL	0.60-1.40 mg/dL
BUN	99 mg/dL	10-26 mg/dL
Urine protein/creatinine ratio, spot	1.7 g/g	<0.2 g/g
Urinalysis		
Protein	100 mg/dL	<29 mg/dL
White blood cell count*	4-10	0-10
Red blood cell count*	Numerous	0-3
Casts	None	None
INR	9.5	2-3
ANA (titer, pattern)	Positive (1:80, speckled)	Negative (<1:40)
c-ANCA	Negative	Negative (<1:20)
p-ANCA (titer)	Positive (1:80)	Negative (<1:20)
MPO Ab	<1.0 U	<1.0 U
PR3 Ab	<1.0 U	<1.0 U
RF	13 IU/mL	<14 IU/mL
Anti-dsDNA Ab	1 IU/mL	<4 IU/mL
Cryoglobulins (cryocrit)	Positive (1.4%)	Negative (0%)
Complement C3	71 mg/dL	54-178 mg/dL
Complement C4	27.7 mg/dL	10.0-42.0 mg/dL
Anti-GBM Ab	<1.0 U	<1.0 U
Syphilis IgG	Nonreactive	Nonreactive
Hepatitis B serologies		
HBsAg	Nonreactive	Nonreactive
HBsAb	Nonreactive	Nonreactive
HBcAb	Nonreactive	Nonreactive
Hepatitis C Ab	Nonreactive	Nonreactive
HIV Ab	Nonreactive	Nonreactive

**Figure 1:**
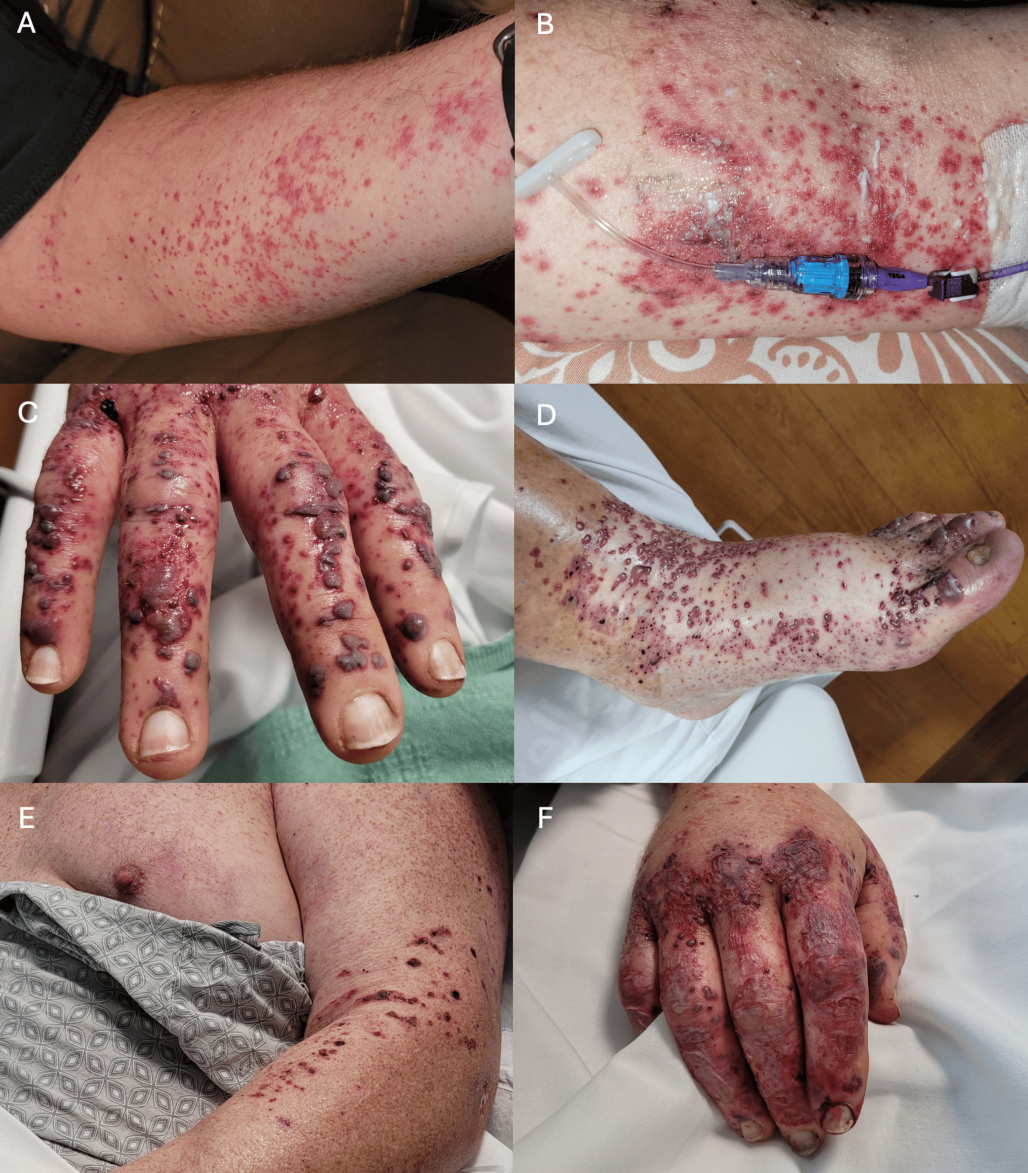
Rash with petechiae, palpable purpura, and hemorrhagic bullae/vesicles A, B: The rash began approximately 11 days after blood cultures returned positive for MSSA, with petechiae across the bilateral upper extremities. Lesions were clustered around the central line insertion site on the left upper extremity; C, D: The lesions then spread to the bilateral upper and lower extremities, including the palmar and plantar surfaces. Petechiae developed into palpable purpura with scattered hemorrhagic bullae and vesicles; E, F: The rash continued to evolve and eventually spread to involve the chest and abdomen. Many lesions became confluent and crusted over. MSSA: Methicillin-sensitive *Staphylococcus aureus*

On exam, there were petechiae and palpable purpura scattered on the bilateral upper extremities, palmar surfaces, axillary folds, ankles, and dorsal feet (Figure 1A-1D). There was initial sparing of the face, abdomen, back, groin, and proximal lower extremities, although throughout his hospitalization, the rash spread to the to involve the proximal lower extremities and lower abdomen. A few vesicles evolved into hemorrhagic bullae, while others became confluent and then crusted over (Figures 1E, 1F). The rash did not involve the mucosal surfaces and was not pruritic or painful. The patient’s exam was suggestive of leukocytoclastic vasculitis, so he underwent skin biopsy and was started on topical steroids while awaiting results. Both warfarin and cefazolin were held due to concern their interaction may be contributing both to rash (as a drug reaction) and elevated INR. He was then started on vancomycin, which was transitioned to nafcillin for treatment of his MSSA.

The patient's renal function initially improved with intravenous fluids, but his serum creatinine began rising again five days into hospitalization. A renal ultrasound was negative for hydronephrosis. Serologic work-up revealed low-titer positive anti-nuclear antibody (ANA) 1:80 (speckled pattern) and perinuclear anti-neutrophil cytoplasmic antibody (p-ANCA) 1:80 with negative myeloperoxidase (MPO) and proteinase-3 (PR-3). He was also found to have positive cryoglobulins with very low quantification (1.4%), which was not felt to be the etiology of his overall clinical picture. He had normal complement levels and negative rheumatoid factor (RF), anti-double-stranded DNA (dsDNA), anti-glomerular basement membrane (GBM), syphilis, hepatitis B and C, and human immunodeficiency virus (HIV) tests.

**Figure 2:**
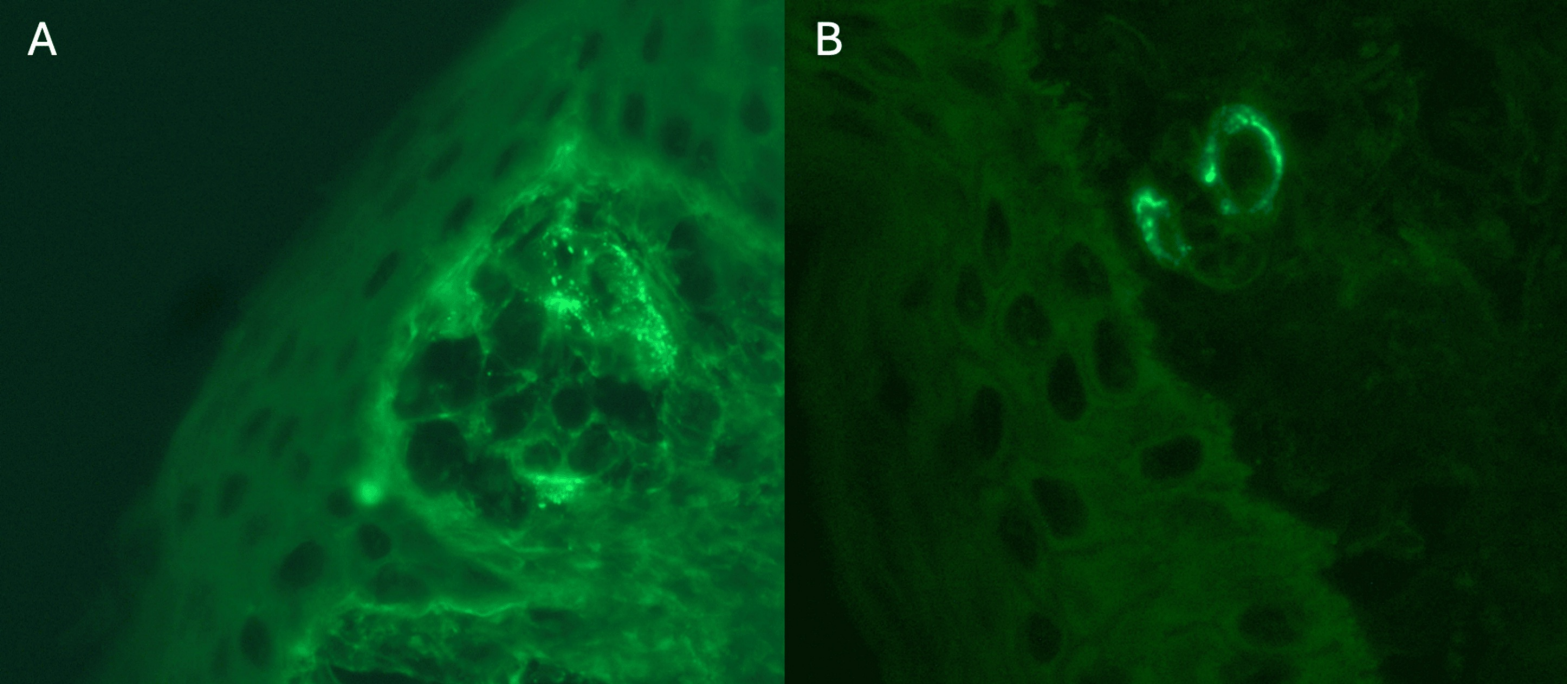
Skin biopsy immunofluorescence There is strong granular deposition of IgA (2A, IgA stain, 40x magnification) and C3 (2B, C3 stain, 40x magnification) within the superficial dermal vessels with C3 showing a slightly stronger intensity than IgA. IgA: Immunoglobulin A

His skin biopsy demonstrated findings consistent with leukocytoclastic vasculitis; immunofluorescence showed granular deposition of IgA (2+), IgM (1+), C3 (2+) in the superficial dermal vessels, raising concern for HSP (Figures 2A, 2B). Given these findings, there was concern for possible HSPN as an etiology for his worsening renal function and was started on oral prednisone 60 mg daily for three days. His INR remained elevated, so his renal biopsy was deferred given the risk of bleeding. He was escalated to pulse dose steroids with IV methylprednisolone 500 mg for three days and then transitioned back to oral prednisone 80 mg but was placed back on IV steroids shortly after due to gastrointestinal (GI) upset. His rash began to improve, and his creatinine peaked at 3.66 mg/dL. Despite improving creatinine, a renal biopsy was obtained to assess for other possible treatable causes and need for escalation of immunosuppressive therapy. His INR decreased to <1.5 with IV vitamin K, but his biopsy was delayed and was performed on hospital day 18. 

**Figure 3:**
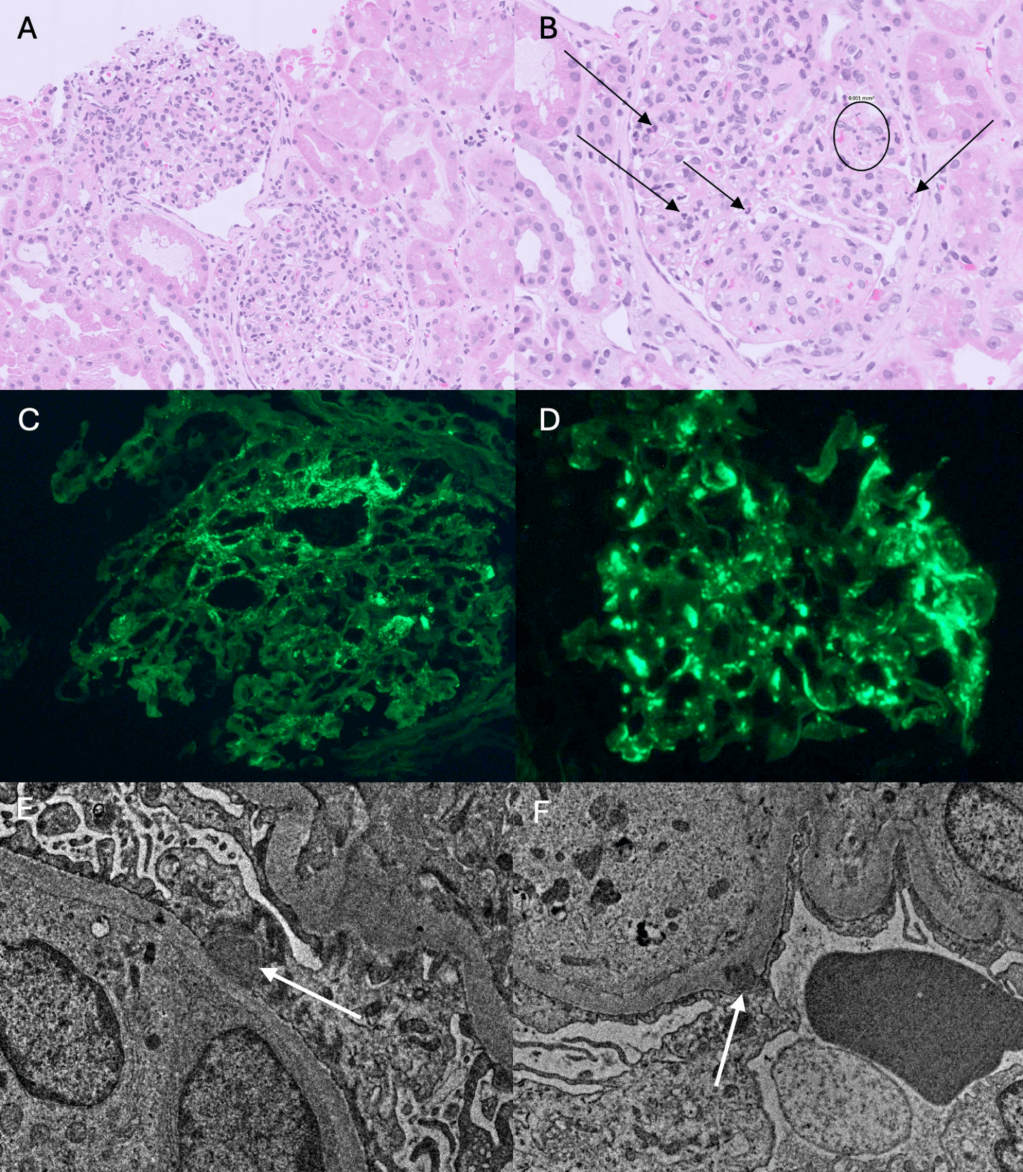
Renal biopsy findings with light microscopy, immunofluorescence, and electron microscopy A, B: The glomeruli show diffuse proliferative features with global endocapillary hypercellularity, mild mesangial proliferation, and exudative features with mildly increased neutrophils (black arrows, 1B) and a focus of karyorrhexis (circle, 1B). (H&E stain, 20x and 40x magnification); C, D: Immunofluorescence shows IgA dominant immunoglobulin deposition in a mixed granular and patchy segmental GBM pattern at 1-2+ intensity (IgA stain, 40x magnification). C3 showed a similar pattern of deposition at slightly brighter intensity (3+) (C3 stain, 40x magnification). There was no significant deposition of IgG, IgM, Kappa, or Lambda. E, F: Along the GBM, characteristic electron dense deposits are seen focally in a subepithelial distribution, also known as a “subepithelial hump” (white arrow, 1E, 8,000x magnification). A few deposits showed invagination into the GBM, appearing as a “cup” type deposit (white arrow, 1F, 8,000x magnification). H&E: Hematoxylin and eosin; IgA: Immunoglobulin A; IgG: Immunoglobulin G; IgM: Immunoglobulin  M; GBM: Glomerular basement membrane

The patient’s renal biopsy demonstrated diffuse proliferative glomerulonephritis with mild exudative features. On light microscopy, 47 glomeruli were examined with 12 exhibiting global sclerosis. Non-sclerotic glomeruli showed diffuse mesangial and endocapillary hypercellularity with most of the glomeruli showing mild exudative features demonstrated by increased intracapillary neutrophils (Figures 3A, 3B). No fibrinoid necrosis or crescents were noted. Immunofluorescence demonstrated granular mixed mesangial and GBM deposition IgA (1-2+) and C3 (3+) (Figures 3C, 3D). There was no significant deposition of other immunoproteins. Electron microscopy showed scattered subepithelial and mesangial deposits with a few subepithelial deposits having a “cup” type invagination as well as a single focal subepithelial “hump” type deposit (Figures 3E, 3F). The overall features raised concern for an IgA-PIGN rather than a typical case of IgAN, especially in the setting of MSSA bacteremia. Given the renal biopsy findings, it was felt that the patient’s overall clinical picture was most consistent with a PIGN provoked by recent MSSA bacteremia with a coincidental minor component of (likely baseline) IgAN, and systemic steroids were discontinued.

The patient’s clinical course was complicated by a post-biopsy retroperitoneal hematoma requiring embolization of a renal artery pseudoaneurysm. His creatinine slightly uptrended after this but slowly returned to close to his baseline prior to discharge and his rash improved. Unfortunately, he was readmitted four days later for recurrent kidney injury and septic shock secondary to enterococcal bacteremia, candidemia, and extended-spectrum beta-lactamase (ESBL)-Klebsiella pneumonia. He then developed a duodenal perforation likely from a duodenal ulcer seen on esophagogastroduodenoscopy. Intraoperative biopsies showed no evidence of intestinal vasculitis. However, immunofluorescence was not performed to evaluate for IgA deposition. His rash returned with greater intensity, and he developed cutaneous ulcerations, particularly on the dorsal hands. A repeat skin biopsy was performed and showed interstitial neutrophilic inflammation and erythrocyte extravasation, most consistent with a septic vasculitis. Immunofluorescence was negative. Despite aggressive treatment with broad-spectrum antibiotics and vasopressor support, the patient developed multi-organ failure and ultimately passed away after transitioning to comfort measures.

## Discussion

IgA-PIGN associated with Staphylococcus infections was first described in 1995 by Koyama et al., who proposed a pathologic mechanism where bacterial endotoxins act as superantigens, binding directly to the T-cell receptor and inducing massive inflammatory cytokine release [3]. Patients typically present with abrupt kidney injury with active urinary sediment, sometimes accompanied by a purpuric skin rash similar to HSP [11]. Patients who have clinical features of HSP and renal injury following staphylococcal infection present a diagnostic challenge because of the overlap in signs and symptoms between HSPN and IgA-PIGN. This distinction is imperative to make because the treatment for one is often ineffective and may even exacerbate the other.

Renal biopsy may be helpful in establishing a diagnosis, and although biopsy findings can often be similar between the two clinical entities, several characteristics are more consistent with IgA-PIGN over HSPN (Table 2). In IgA-PIGN, light microscopy typically demonstrates diffuse endocapillary and mesangial hypercellularity as well as infiltrating polymorphonuclear and mononuclear cell infiltrates within capillary loops. Immunofluorescence commonly shows granular IgA and C3 deposition in a mixed mesangial and GBM distribution, with C3 typically showing stronger intensity deposition over IgA. Electron microscopy shows primarily mesangial electron dense deposits with a minority of cases showing focal large subepithelial deposits (“humps”) [4-9,11-14]. HSPN renal biopsy findings can appear similar to IgA-PIGN in that it often shows mesangial proliferation with granular IgA and C3 deposition [12]. The strong staining of C3 over IgA, subepithelial deposits, and diffuse endocapillary hypercellularity with inflammatory neutrophil infiltration seen in our patient support a diagnosis of IgA-PIGN over HSPN [4,8,11,12].

**Table 2:** Histopathologic findings of IgA-PIGN vs. HSPN IgA: Immunoglobulin A; IgA-PIGN: IgA-dominant postinfectious glomerulonephritis;  HSPN: Henoch-Schönlein purpura nephritis

Feature	IgA-PIGN	HSPN
Mesangial proliferation	Present	Usually present
Endocapillary hypercellularity	Marked and diffuse, often with neutrophilic infiltration	Less prominent
Immunofluorescence pattern	Granular C3 and IgA deposition	Granular C3 and IgA deposition
Immunofluorescence staining dominance	C3 > IgA	IgA > C3
Location of deposits on electron microscopy	Mesangial ± subepithelial “hump”-shaped deposits	Predominantly mesangial

Patients with IgA-PIGN can commonly present with skin findings that can mimic HSP such as palpable purpura with skin biopsy showing leukocytoclastic vasculitis and vascular IgA deposition [4,11,14,16,18,19]. However, there are often other clinical features that may be helpful in establishing the correct diagnosis. The presence of renal failure concurrently or shortly after a pyogenic infection, older age of onset, comorbid diabetes, and heavy proteinuria strongly favor IgA-PIGN, while HSPN typically affects younger patients, follows a URI, and rarely leads to renal failure [5,10-12,20]. Although other forms of PIGN are typically associated with hypocomplementemia, our patient demonstrated normal serum complement levels and this phenomenon has also been documented by others [9,11,20].

Making the correct diagnosis in patients such as ours is important because the treatment pathways often diverge. While HSPN typically improves with steroids, the mainstay of IgA-PIGN is treatment of infection. Contrary to classic PIGN, IgA-PIGN does not necessarily resolve with full treatment of the infection. Therefore, cases of IgA-PIGN are often treated with corticosteroids, particularly when the diagnosis is not entirely clear, or in those who fail to respond to antimicrobial therapy alone [8,12]. Our patient initially presented with a purpuric rash and with only mild kidney injury that was initially attributed to dehydration. However, as his renal function deteriorated, the suspicion for HSPN increased, especially in the setting of a skin biopsy consistent with IgA vasculitis, prompting us to begin steroids. It was not until his kidney biopsy demonstrated possible IgA-PIGN did we suspect this as a unifying diagnosis. Although his creatinine did improve with steroids, it is unclear whether this was a direct result of immunosuppression or the antibiotic treatment he was also receiving at the time. Furthermore, he ultimately developed complications that may have been related to or exacerbated by steroid exposure including infection, intestinal ulceration, and death.

## Conclusions

As evidenced by our case, a high degree of clinical suspicion is necessary for the diagnosis of IgA-PIGN in Staphylococcal infection due to its ability to mimic HSPN. Although renal biopsy was helpful in this case, it may not always be possible to obtain one or it may be significantly delayed due to procedure risks, such as the concern for bleeding in our patient. Without a kidney biopsy, our initial suspicion of HSPN due to the patient’s renal injury, purpuric rash, and IgA deposition on skin biopsy resulted in him receiving weeks of likely unnecessary high-dose steroids, increasing his risk of morbidity and mortality. Beyond obtaining a renal biopsy, being aware of clinical signs that may indicate a diagnosis of IgA-PIGN over HSPN is imperative, including the type of concurrent infection and urinalysis findings. We hope this case will help future patients receive the appropriate treatment for their condition and avoid the complications of misdiagnosis.
